# Rapid review of factors influencing dietary behaviors in Fiji

**DOI:** 10.3389/fnut.2023.1164855

**Published:** 2023-08-09

**Authors:** Benjamin Boxer, Ursula Trübswasser, Viola Lesi, Asaeli Naika, Pradiumna Dahal, Sonya Sagan, Kshitij Joshi, Ana Irache, Pragya Singh, Devina Nand, Ateca Kama, Alvina Deo, Sophie Goudet

**Affiliations:** ^1^Nutrition Research, Dikoda, London, United Kingdom; ^2^UNICEF Pacific, Pacific Islands, Suva, Fiji; ^3^School of Public Health and Primary Care, College of Medicine, Nursing and Health Sciences, Fiji National University, Suva, Fiji; ^4^Ministry of Health and Medical Services, Suva, Fiji; ^5^National Food and Nutrition Centre, Suva, Fiji

**Keywords:** food environment, obesity, malnutrition, social influences, unhealthy foods

## Abstract

**Introduction:**

In Fiji, multiple burdens of malnutrition including undernutrition, overweight/obesity, and micronutrient deficiencies coexist at the individual, household, and population levels. The diets of children, adolescents, and adults are generally unhealthy. The objective of this review was to understand how the dietary behaviors of children, adolescents, and women in Fiji are influenced by individual, social, and food environment factors.

**Methods:**

This rapid review was conducted to synthesize existing evidence, identify research gaps in the evidence base, and make recommendations for future research. The Cochrane Rapid Reviews Methods and the updated guideline for reporting systematic reviews were used. The search strategy for this rapid review was based on the Population Context Outcome [P(E)CO] framework, including search terms for population (children, adolescents, and adults), context (Fiji), and outcome (dietary behaviors). Searches were conducted in PubMed, Scopus, PsycINFO, and Google Scholar.

**Results:**

The 22 studies included in this review identified different factors influencing dietary behaviors in Fiji. Individual preferences for processed and imported foods, especially of younger generations, and social dynamics, especially gender norms and social pressure, to serve meat and overeat appeared to be prominent in driving dietary habits. The ongoing nutrition transition has led to increasing availability and affordability of ultra-processed and fast foods, especially in urban areas. Concerns about food safety and contamination and climate change and its effect on local food production also appear to influence dietary choices.

**Discussion:**

This review identified different dynamics influencing dietary behaviors, but also research gaps especially with regard to the food environment, calling for an integrated approach to address these factors more systemically.

## 1. Introduction

Globally, diets are rapidly shifting from traditional diets to highly processed high-energy diets, which has resulted in both undernutrition and obesity occurring simultaneously in many countries (1). WHO reported that this double burden of malnutrition contributes to 78% of deaths in middle- and low-income countries (2). In Fiji, multiple forms of malnutrition coexist at the individual, household, and population levels. The prevalence of underweight in the adult population of Fiji was 1.7% with overweight at 65.6% and obesity and high blood pressure at 33.4 and 21.4%, respectively, with similar rates of overweight and obesity reported in adolescents (34.0 and 12.8%) (3).

Diets of older children, adolescents, and adults in Fiji are generally unhealthy, characterized by low intake of fruits, vegetables (particularly very few consume indigenous and traditional varieties of starchy crops and green leafy vegetables), whole grains, legumes, milk, and nuts/seeds, and high intake of sodium and sugar-sweetened beverages (SSBs) (4). In Fiji, as well as in other Pacific Island Countries (PICs), these unhealthy dietary trends have been associated with the rapid food system transformation that has taken place over the past decade (5–8).

A review of the determinants of overweight and obesity in PICS concluded that environmental-related factors and socio-cultural-related dynamics play an important role (9). However, no comprehensive review of the literature exists on what influences dietary behaviors in Fiji. Few studies have identified potential factors such as price, convenience, availability, accessibility, healthfulness, food safety, taste, and familiarity that influence the dietary behaviors of Fijians (10). Due to the diversity in cultural and ethnic backgrounds of the population and the geographical location of communities across the country, factors influencing dietary behaviors can be complex. Furthermore, identifying and exploring these influencing factors can reveal a new perspective to addressing the double burden of malnutrition in Fiji. The aim of this review was, therefore, to understand how feeding practices and dietary behaviors of children, adolescents, and women in Fiji are influenced by individual, social, and food environment factors to identify the state of awareness among target groups and knowledge and identify gaps.

## 2. Methods

This rapid review was conducted to synthesize existing evidence, identify research gaps in the evidence base, and make recommendations for future research (11). Rapid reviews are an approach to synthesize information to inform decision-makers on time. The Cochrane Rapid Reviews Methods Group was used as guidance for the rapid reviews (12) and the updated guideline for reporting systematic reviews (13).

### 2.1. Conceptual framework

**Figure 1:**
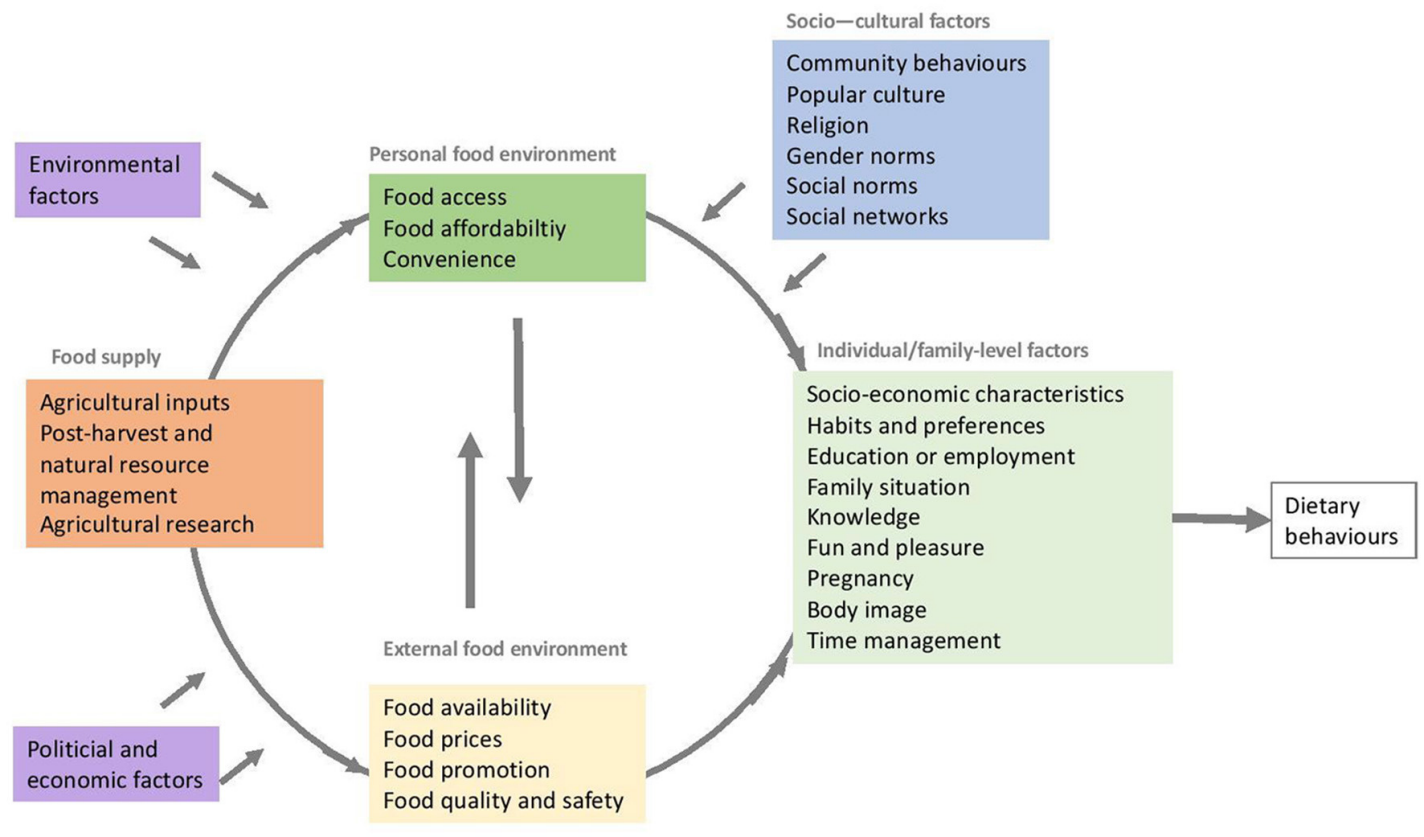
Framework on factors influencing dietary behaviors of children, adolescents, and women [adapted from Osei-Kwasi et al. (14), Raza et al. (15), Story et al. (16), and Turner et al. (17)].

For this review, we used a framework to develop the search terms and the coding structure for data extraction and to guide the analysis (Figure 1). The framework was based on existing frameworks and theories that conceptualized how different factors influence the dietary behaviors of children, adolescents, and women (14, 15, 17). Based on the “best fit” framework synthesis approach, we have deconstructed the elements of different existing frameworks to develop a framework that fits the purpose of this review (18). The Innocenti food systems framework was used as a basis (15), which covers the factors in the personal and external food environment (17), food supply, as well as external drivers. As part of the drivers, this review focused on political, economic, and environmental systems, which were reported and discussed as “macro-level” factors in line with the socio-ecological framework of Story et al. and Osei-Kwasi et al. (14, 16). Since the individual and social level concepts were addressed more comprehensively in other frameworks (14, 16), the framework developed by Raza et al. (15) was further complemented with additional concepts related to individual and socio-cultural factors.

### 2.2. Search strategy

The search strategy was developed based on the Population (Exposure) Context Outcome (P(E)CO) framework (19), combining terms for population (any age or population group), context (Fiji), and outcome (dietary behaviors). The following search terms were used in PubMed (diet^*^[All Fields] OR nutrition[All Fields] OR meal^*^[All Fields] OR eat^*^[All Fields] OR Diet, Food and Nutrition[MESH Terms] AND (Fiji[All Fields] OR Fijian^*^[All Fields]) and adapted for the other databases. Searches were conducted in PubMed (http://www/ncbi.nlm.nih.gob/pubmed), Scopus (https://www.scopus.com), and PsychINFO (https://www.ebsco.com/products/research-databases/apa-psycinfo) using the list of key search terms. Searches were limited to human studies and English language publications. Gray literature and unpublished literature were sought through Google Scholar, where the first 10 pages were screened for titles. Additionally, references from reviewed articles or those brought to the attention of the authors by members of the team were also considered. All identified references were imported into Mendeley and then to Excel, where title and abstract screening was conducted.

### 2.3. Eligibility criteria

In terms of the population group, all age groups were included. Studies that included populations defined by specific diseases or health conditions such as heart disease, hypertension, diabetes, dementia, coeliac, anorexia, preterm birth, HIV, and depression diagnosis, or populations being treated in a clinical therapeutic setting, hospital settings, or extremely niche populations with specific nutritional requirements such as professional athletes were excluded. The exposure of interest was any influencing factor related to the individual level, such as socio-economic background, knowledge of the individual, social-level dynamics referring to family and peers, and factors in the personal and external food environment, as well as on the macro level such as political, economic, and environmental factors. The outcome of interest of our review was dietary behaviors, comprised of consumer behaviors (acquisition/preparation/storage/meal practices) and diets (quality/quantity/safety) (20). Study designs included in the review were qualitative, quantitative, as well as mixed methods approaches. Purely descriptive studies not identifying any associations between influencing factors and dietary outcomes were excluded.

### 2.4. Screening and study selection

The references identified through the searches were imported into Excel, where duplicate records were removed. BB and UT conducted the title and abstract screening in duplicate. The full-text screening was done by two reviewers (UT and BB), and 20% were double-screened by the same reviewers. Justifications for exclusion based on the eligibility criteria were recorded at the full-text screening stage. Any disagreements arising at any stage of the screening process were resolved via discussion between reviewers.

### 2.5. Data extraction and synthesis

The following data were extracted in Excel: title, author, year, geographic division, or island of Fiji (northern, western, central, and eastern), setting (rural and urban), population (age), sex (male/female), sample size, study design, outcome (type of dietary behavior), level of influence (individual, food environment, and socio-cultural), type of influencing factor based on the framework concepts (individual level, socio-cultural dynamics, personal and external food environment, food supply, and environmental and economic factors), and main finding. Data extraction was conducted by BB and extraction of all articles was checked by UT. Data from all factors were grouped by the concepts of the framework and screened for key themes. Illustrative quotations and the number of relevant references were summarized.

## 3. Results

### 3.1. Study characteristics

**Figure 2:**
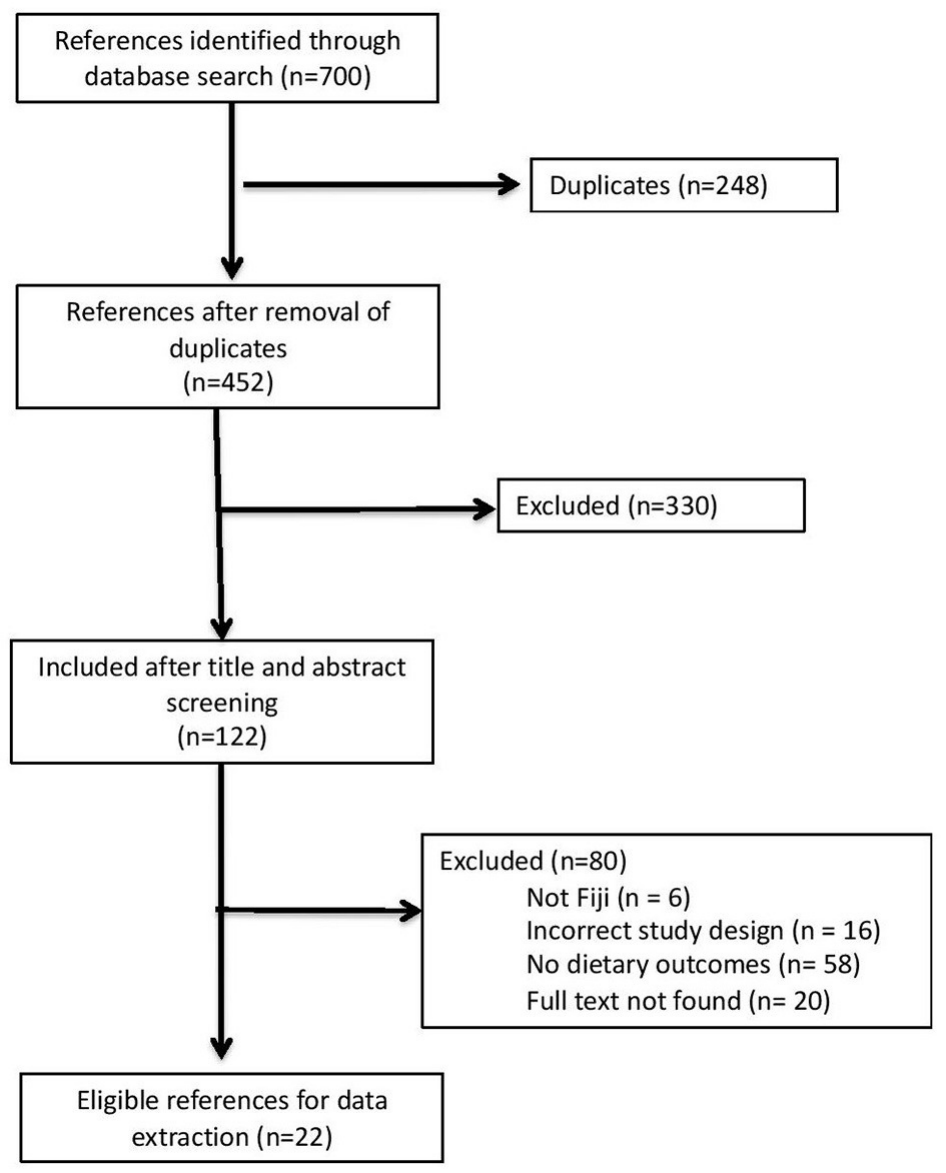
PRISMA diagram showing the systematic screening process.

The search identified 700 references. After de-duplication, title, abstract, and full-text screening, this review resulted in 22 qualitative, quantitative, and mixed-methods studies (Figure 2). The included studies were conducted between 1984 and 2022. Most studies were conducted with adults above 18 years (*n* = 8), children or adolescents aged under 19 years (*n* = 8), and adolescents and adults from 15 years and above (*n* = 3), with three studies not reporting the age of participants. Only one study was conducted with caregivers of children under 5 years of age. Sample sizes of studies ranged from 14 to 6,871 participants. Sixteen studies were conducted with male and female participants and six with women only. Twenty-one studies looked at diets as an outcome, which was either measured as fruit and vegetable intake, dietary diversity, or food consumption in general (Table 1).

**Table 1:** Key characteristics of all included studies (*n* = 22).^*^

**References**	**Setting**	**Design**	**Sex**	**Age (range and/or mean)**	**Sample size**	**Individual/ family level**	**Socio-cultural level**	**External FE**	**Personal FE**	**Macro level**
Bhagtani et al. (21)	Urban and rural	QUANT, CS	M, F	> 15 y	186	x	-	-	-	-
Buksh et al. (22)	Urban	QUAL, CS	F	23–48 y	15	x	x	-	x	-
				Mean: 36.3 y						
Darfour-Oduro et al. (23)	Not reported	QUANT, CS	M, F	13–17 y	1,664	-	-	-	-	x
Guell et al. (24)	Urban and rural	QUAL, CS	M, F	Not reported	76	x	x	x	x	x
Hawea et al. (25)	Urban	QUAL, CS	M, F	≥18 y; 6 m to 5 y	72	x	x	-	x	-
Haynes et al. (26)	Urban and rural	QUANT, CS	M, F	≥15 y	186	-	-	x	x	x
Henrich et al. (27)	Rural	QUANT, CS	F	Not reported	75	-	x	-	-	-
Hidalgo et al. (7)	Rural	QUANT, CS	F	Not reported	64	x	x	x	x	x
Katz et al. (28)	Not reported	QUANT, CS	M, F	7–50 m	35	x	x	x	x	-
McCabe et al. (29)	Not reported	QUANT, CS	M, F	Mean: 16.2 y (Fijians)	1,091	-	x	-	-	-
				15.2 y (Indo-Fijians)						
McKenzie et al. (30)	Peri-urban and rural	QUAL, CS	M, F	Mean: 49 (women) 44 (men) y	46	x	x	x	x	x
Morgan et al. (31)	Urban	QUAL, CS	M, F	>18 y	57	x	x	x	x	-
Neill et al. (32)	Urban and rural	QUANT, CS	M, F	5–16 y	578	x	-	x	-	x
O'Meara et al. (4)	Rural	QUANT, CS	M, F	>18 y	161	x	x	x	x	-
Singh et al. (33)	Rural	QUAL, CS	M, F	> 18 y	14	x	x	x	x	x
Taylor et al. (34)	Urban and rural	QUANT, CS	M, F	≥20 y	846	-	-	x	-	x
Thompson-McCormick et al. (35)	Peri-urban/rural	QUANT, CS	F	Mean 16.7 y	523	-	x	-	-	-
Toren et al. (36)	Not reported	QUAL, LONG	M, F	< 14 y	45/47/100	x	x	x	x	-
Waqa et al. (37)	Not reported	QUAL, CS	M, F	16–18 y	48	x	x	-	x	-
Wate et al. (38)	Peri-urban	QUANT, CS	F	13–18 y	6,871	x	-	-	-	-
Withrow-Wong et al. (39)	Rural and urban	QUANT, CS	F	Mean 43.6 y 18–87 y	68	x	-	-	-	-
				18–87 y						
Witter et al. (40)	Urban and rural	QUANT	M, F	>15 y	-	x	x	x	x	x

^*^CS, cross-sectional; LONG, longitudinal; QUAL, qualitative; QUANT, quantitative; F, female; M, male; SES, socio-economic status; FE, Food environment.

### 3.2. Main findings by framework category

#### 3.2.1. Individual/family-level

##### 3.2.1.1. Family dietary habits and preferences

Four studies found that family members preferred processed foods over traditional home-grown foods, and this influenced what parents purchased and cooked with taste being the main influence on dietary intake rather than nutritional content (22, 24, 30, 33). The following quote from a study participant highlights how preferences differ between generations:

“*I went fishing last week at the creek. I caught a lot of tilapia but my eldest son doesn't eat tilapia he went bought tinned tuna for himself* ” [Male, adult] (24).

In addition, mothers tended to try to please their children through food offerings, and it was emphasized that any additional money the family had was often spent on processed food due to the changing preferences for such foods. A mother said:

“*My kids love the blue packet Twisties [extra-cheese-flavored snack] and whenever we have extra [money] so then we buy some”* [Female, adult] (22).

Despite trying to please their children, parents appear to have a positive influence on fruit and vegetable consumption (33) and showed concern about the increased preference for processed foods and encouraged their children to reduce their consumption of unhealthy foods such as recommending them to “*cut down on oily foods*” (30).

In addition to individual family member's preferences, the opinions of other family members were reported to influence food preferences with a particular emphasis placed on parents and grandparents (25, 37). The dietary advice from parents was both positive as well as negative and centered around advice relating to losing weight (37). The influence of grandparents was often seen as positive. However, one study described this influence as negative, as a mother said:

“*I ban my children from going to see their grandparents because every time they go, they come back with some junk foods like bongo, and lollies*” [Female, adult] (25).

The differing attitude toward dietary intake among children, parents, and grandparents may reflect generational changes in dietary preferences with younger people eating less traditional foods (30, 33).

##### 3.2.1.2. Individual dietary habits and preferences

There were four main themes under individual diet/habits/preferences. The first theme is related to the ease of preparation and consumption of processed food with ample availability, taste preference, and ease of access (26, 33). The second theme referred to the shift in family preferences toward processed foods and relates to individual preference for processed foods rather than meals prepared using traditional ingredients and vegetables (4, 25, 31, 32, 37). Participants reported a preference and consumption of highly processed and imported foods such as high-sugar foods or drinks, cookies, and fried foods and vegetables (4, 25, 31, 32, 38).

##### 3.2.1.3. Knowledge

The perceived benefits of homegrown/traditional food were themes that participants referred to in four studies. Participants of included studies believed that “*healthy foods are the foods that we grow*” [Female, adult] (24) which would include fish cooked in coconut milk and vegetables (30, 37), and there was a significant positive correlation between the consumption of traditional starches and perceived health (39).

Participants also seemed to be aware of government guidelines to reduce the burden of NCDs and the link between diet and disease with sugar, salt, and fat being identified as key nutrients of concern (24, 31):

“A *lot of high blood pressure, diabetes.... heart attack, kidney failure and lungs, this is simply because we are not taking a lot of locally produced food, like the vegetables. We eat a lot of processed food from the shop”* [Female, Adult] (31).

Knowledge regarding the relation between diet and diseases, such as diabetes, heart disease, and poor dental health (30), and individual diagnosis of such diseases caused some participants to change their diet:

“*Before we were eating a lot of root crops, but when we were diagnosed with some sickness we are trying to adhere to doctors' advice to cut down on certain foods”* [Female, adult] (24).

In addition to being able to identify the link between diet and disease, most participants could identify what constituted a healthy diet (37) and a balanced diet “*like eating fruit (and) three different food types”* [Student] (30, 37). Mothers were also aware that the dietary habits that children pick up during their childhood could continue into adult life, negatively impacting long-term health outcomes:

“*It's all upon the mothers to teach the children [about healthy eating] at home. The type of vegetables and fruits you give them, they'll eat it. If you won't—if you just force them or just give them the junk foods, they'll just be trained on that”* [Female, adult] (31).

Although participants were aware of the negative health effects of consumption of nutrient-poor and energy-dense foods such as high in fat, sugar, and salt foods, they were less aware of the relation between the quantity of food consumed and health issues (7).

##### 3.2.1.4. Fun and pleasure

Participants of one study highlighted that food was an indicator of generosity, love, happiness, and affection for others with the phrase “*kana meda bula”* (eat to live) being a commonly used phrase in Fiji (22):

“*Kana meda bula! The more you eat, the more you live, in fact the better you live [Laughs]. You leave your, what you say, your diet, or worries about health and enjoy the vibe, the environment, and the company [laughs]. Basically, enjoy now and worry about those things later. And so, you get tempted, I mean, who wouldn't?”* [Female, adult] (22).

##### 3.2.1.5. Pregnancy

Only one study reported data related to overeating during pregnancy. Pregnant women in this study were encouraged to overeat during pregnancy for the wellbeing of the child, but then lose weight post-partum:

“*I remember when, when I got pregnant, in my early months of pregnancy I was still skinny, and oh my elders were telling me you're not healthy the baby is suffering, you need to eat a lot, and I'm thinking, “What? What does that have to do with the baby?” … when I was pregnant with my daughter. They like, “Eat, eat, eat!” and then when I was breastfeeding, “Eat, eat, eat!” and after I had my daughter and I weaned off my daughter, they started “Stop eating, stop eating, stop eating!”* [Female, adult] (22).

##### 3.2.1.6. Time management

Adult participants in three studies in the review reported consuming fast food due to a lack of time to tend to agricultural activities (7) or to prepare food (22, 30):

“*Nowadays, we only access fast food or take away foods, because we have no time to cook at home, so we go for Pizza and other fast foods”* [Female, adult] (30).

Children also stated that lack of time impacted their dietary intake leading to them skipping breakfast (37).

#### 3.2.2. Socio-cultural factors

Our review identified four socio-cultural factors: community habits, social norms, gender norms, and social networks.

##### 3.2.2.1. Community behaviors

Under community habits, our review identified two main themes: the community's perceptions of traditional compared to modern foods and the importance of eating together at social gatherings. Ten studies included data related to community habits. Fruits and vegetables were perceived as essential components of the traditional diet, but transitions to more unhealthy food consumption and preparation methods were described (31, 35, 40). Changes in preparing food referred to the use of oil and frying of food (7). While dietary behaviors associated with Westernization such as breakfast skipping were perceived as unhealthy, traditional foods were perceived as healthy (24, 35, 40):

“*Healthy foods are the foods that we grow. It makes our body healthy like cassava. Our forefathers used to have tea with cassava”* [Female, adult] (24).

The social aspect of eating was identified as another key theme. Six studies discussed how eating together with family, friends, and neighbors at special cultural and religious celebrations is an important part of iTaukei culture (22, 25, 29, 30, 33, 36). The food served at these gatherings was served in large amounts and usually contained meat and little fruits and vegetables (22, 25, 30):

“*In our culture, we present to funerals, weddings, birthdays, or other functions there are a lot of meat like pig, beef, chicken, fish, and dalo, cassava or yams”* [Female, adult] (25).

The success of an event was judged by an abundance of food, especially meat, which was associated with “generosity, happiness, love, affection and the buying-power of the host” (22).

Even though study participants were aware of the benefits of fruits and vegetables, in social gatherings, it was expected to serve meat to show the status of the family since serving vegetables might mean that the family cannot afford meat. The preparation of such foods was, therefore, not necessarily in line with what people usually eat and might interfere with healthy dietary behaviors (22, 33), as this iTaukei participant explained:

“*Yes, like you know how we alternate between veggies and meat or fish every day? And we enjoy simple meals, generally boiled leafy greens? All that goes out the window when we have people over [laughs]. You have to make something special and maybe a few types of dishes with meat and generally more rich food like add lolo (coconut cream) to the dishes. Like if we have a lovo, it's a lot of meat, a lot of coconut cream. So, the meal does become very unhealthy. In fact, the meal becomes exactly what I discourage at home”* [Female, adult] (22).

##### 3.2.2.2. Gender norms

Traditional gender roles of women preparing and serving food, men receiving preferential provision of food, and changes related to these roles were identified as the main themes in seven studies. The traditional role of women was perceived as the one having to prepare and serve food for the family and take care of the health of the family and domestic responsibilities, while men were seen as the ones responsible for earning money to feed the family (28, 30, 33). Women preparing food for the family was associated with women's love and care for their families (30). Mothers were also perceived as having a more positive influence on the diets of children compared to men, as this mother reflected:

“*When I buy, I look for fruits unlike my husband, he likes to buy something cheap, especially junks when they sell for a cheap price. I tell him, our son will get sick and it is more expensive being sick than eating fruits and remaining well, he doesn't see it that way*” [Female, adult] (25).

The perceived role of men as the breadwinner was also associated with the need to be fed first and receive more food of higher quality (22, 30, 33, 36). This practice of giving preference to the man when it comes to food within the household was perceived as a “form of showing respect” (30).

“*Men are generally encouraged to eat more, because men are heads of family, they sort of take the top place and they are expected to do hard work*” [Female, adult] (22).

Men are not only receiving food first but they also get the best and largest portions, while the women wait for their husbands to finish eating before they start eating whatever is left (30, 33, 36). Eating the leftovers could lead to binge eating habits as one study participant said:

“*Normally that's culture. That is the Fijian culture. Men used to eat first and then the women will eat later but normally eating later that means everything that's left they are going to have it*…” (Adult) (33).

However, three studies discussed how these roles might be changing with men doing less of the hard labor such as farming or fishing and engaging in more sedentary activities while women increasingly work out of the house (7, 22, 30). Despite these changes, men seem to get preferential treatment when it comes to food allocation:

“*Men are not doing their work […] before they used to plant cassava and we always have plenty of it, only the elders used to do that, and we just eat them (the cassava). Now, the men are sleeping*” [Female, adult] (30).

Study participants reflected on these changes, requesting a change in food being allocated since women work just as hard as men (30).

The fact that more women work outside the house limited their time to prepare food at home, making families more dependent on processed food. Equally, a study reported that men who still work in agricultural production focused on cash crops, which also forces the family to rely more on purchased processed foods (7).

##### 3.2.2.3. Social norms

This review identified seven publications discussing social norms related to social pressure to overeat at social and religious gatherings as well as specific foods and larger body sizes being associated with higher social status. Large gatherings which are an important part of iTaukei culture encourage overeating since guests are encouraged to eat more and feel the social pressure to show respect to the host (22, 25, 29, 36).

“*In our culture, if anyone offers food, it's kind of disrespectful to decline. Like it's a bit rude and times you feel that when you decline you are giving the message that the food isn't good either. So, it both sides [sic] and ends with people eating way more than they should*” [Female, adult] (22).

The pressure is also felt by the hosts who are expected to serve plenty of food to present themselves as generous hosts.

“*And so, we keep refilling the bowls on the serving table and we encourage our guests to eat well. It looks bad if a serving bowl is empty or if run out of food. So, when you cook or cater, you always make sure there's leftovers”* [Female, adult] (22).

Three studies reported on food taboos (27) and certain foods being associated with high social status, such as meat, seafood, and fast food, while vegetables were perceived as poor people's food (22, 25). Owning livestock was also considered a privilege (25). Eating out of the home, especially fast food, was perceived as something that could only be done by rich people or at certain moments when people had more income (22, 24, 40):

“*So, it's only when we can afford it. I think people only eat out when they can afford it so they have the money, they are rich, they can afford to go out for burgers and chicken and chips, pizza and all those kinds of food*...” [Female, adult] (22).

Good social status was also associated with larger body size and the size of children, or a man reflected how well a woman took care of her family (22, 30, 33, 40).

#### 3.2.3. Personal food environment

##### 3.2.3.1. Food affordability

The affordability of food was the central theme in eight studies (22, 24, 25, 30, 31, 33, 40). Affordability could determine what people purchase and eat, limiting the options people have. Foods that are filling such as carbohydrate-rich foods like rice or bread were prioritized over fruits and vegetables when people faced financial challenges (30). Certain foods such as fruit and vegetables, tin fish, and packed instant noodles were considered more affordable, while fresh meat, fish, seafood, and fast food were seen as more expensive (22, 25).

“*So, it's really the price. I always opt for tuna, tinned fish, or sausages because you can. spread it to a few meals. Mel: I will do tinned fish and tuna, and corned mutton and, especially, sausages because we can't afford fresh beef and pork*” [Female, adult] (22).

A study in a rural setting showed that people relying on lower incomes were forced to buy cheaper options at small stores in the villages or get takeaway food from the cities where they sold the vegetables they grew. However, consuming homegrown food was seen as an affordable way to get fruits and vegetables for farmers or people who had home gardens (24). Consumption of food from home gardens was found to be associated with higher fruit consumption (24, 26).

##### 3.2.3.2. Food access

Increase access to unhealthy foods to both urban and rural populations was reported by three studies (7, 30, 33). Improved infrastructure of roads and transport made food, especially unhealthy ultra-processed food, more accessible to people living in villages (30, 33). At the same time, access to traditional and homegrown food was decreasing with the effect of climate change on food production (30).

#### 3.2.4. External food environment

##### 3.2.4.1. Food availability

Food availability was also associated with the types of food vendors raised by participants in 4 studies. A study found that more than half of the participants purchased food from supermarkets more than once a week (21). However, for people affected by poverty, smaller shops were more accessible and therefore an important source of food. However, small shops were also associated with food insecurity and lower dietary diversity, higher intake of sugar-sweetened beverages, and red and processed meat (21, 26). While healthy, minimally processed food appeared to be available to people, the omnipresence of processed foods tempted people to buy them (30):

“*There is a lot of processed food. We should eat the food that lives free, like taro leaves. It is around us, the food that we supposed to eat and then we are going to the shop to buy tinned fish and things like that*” [Male, adult] (30).

The availability of different foods was a theme identified in two articles (30, 37). The food available at schools such as snacks high in sugar, salt, and fat influenced students to buy them even if canteens provided curries and rice (37). Study participants expressed concerns about the availability of unhealthy snacks in and around schools, which are even sold by teachers, which puts parents in a difficult situation (30).

##### 3.2.4.2. Food promotion

Two studies provided data on widespread food advertising, especially of unhealthy, processed food, which influences especially rural people to buy unhealthy food (33, 40).

##### 3.2.4.3. Food quality and safety

Study participants of 3 studies expressed concerns about chemical and pesticide contamination of local foods (24, 30, 31). These concerns were related to the perception of poorly regulated pesticide use on local agricultural produce and affected the taste and the quality of food (24, 30).

“*I've noticed that most of the farmers they are using a lot of chemicals on chauraiya (amarnath leaves). Once I bought it from the market…. We could smell the chemical … I refused to eat chauriya. Before it used to be my favorite”* [Male, adult] (31).

However, imported processed foods were also associated with a fear of chemicals. Buying food from trusted vendors and avoiding tinned and frozen food was an approach participants took to avoid chemical threats from these foods (31).

#### 3.2.5. Food supply

Six studies addressed the issue of food supply and how it may affect consumption (7, 24, 30, 32, 33, 40). Access to land was limited due to far distances, even for people living in villages. This appears to be a limiting factor for people to produce their own food, forcing them to purchase food from shops, even if people owned a farm (24). However, in rural areas, access to land was still better than for urban populations. Neill et al. found that 75% of rural and 47% of urban populations had access to food products for home consumption (32). Besides the distance and general lack of access for people living in urban settings, they also mentioned a lack of time and interest in home gardens (24, 32).

[…] P: *Where I'm renting now, we don't have that luxury to plant what you want to so you just gotta buy everything. From market or the supermarket, sometimes it's imported. “previously we use to get food from the garden however now we seem to be buying a lot*” [Female, adult] (24).

Another important mechanism limiting food production mentioned in a study was the lack of government subsidies, equipment, and knowledge transfer for local production of fruits and vegetables (33).

#### 3.2.6. Political and economic factors

Global trade appeared as an important issue reported in three studies (7, 24, 31). Imported foods, especially white flour, white rice, and added sugars, were perceived as widely available, but with mixed impacts on health. Some participants associated imported food with unhealthy food and negative health impacts related to obesity and non-communicable disease. However, some participants considered local food to be equally unhealthy than imported food:

“F: *Does it matter if they are local or from abroad? Any difference? P: I don't believe so. Maybe even unhealthier? F: Fiji products have a lot of oil content, [same brand name of tinned fish] has a lot of oil, overseas product is dry instead*” [Male, adult] (24).

Food imports were also mentioned as a positive influence on the availability of especially fruits and vegetables, such as apples and carrots, which would not be available throughout the year if they were not imported (34).

Urbanization or the difference between urban and rural diets was addressed in four studies (21, 32, 34, 40). Rural populations were reported to consume higher quantities of energy, especially in the iTaukei population, such as traditional root crops, which were lower in urban diets (40). Rural diets were also described as less processed and lower in diversity, and higher in carbohydrates but lower in fats than urban diets (21, 32, 34).

#### 3.2.7. Environmental factors

Four of the included studies in this review highlighted that climate change is making it harder for individuals to plant and grow crops. Participants stated that previously they were able to grow crops year-round but now it was too dry in some months, and in addition to inconsistent rainfall, natural disasters were affecting the supply chain leading to an increased reliance on processed, packaged foods. Studies emphasized that increasing temperatures and limited water availability were adversely affecting their ability to grow fruits and vegetables (7, 32). In addition to the increasing temperatures and limited water availability, rising sea levels were mentioned regarding lower land availability for growing crops (30). Cyclones were also reported to impact market access and limited food availability (7, 30). These natural disasters impacted food prices and consequently individuals' food choices (31).

## 4. Discussion

This rapid review aimed to synthesize existing data on factors influencing diets in Fiji. Individual and social dynamics as well as the ongoing nutrition transition, food safety concerns, and climate change appeared to be prominent in driving dietary habits with a preference for processed and imported foods with social norms around feasting leading to overconsumption.

On the individual/family level, we found good knowledge related to traditional, local food being healthy and the need to reduce salt, fat, and sugar. However, changing preferences of younger generations were linked to more modern, processed foods compared to older generations. These dietary shifts, defined as the “nutrition transition” (1), were also described in a review of qualitative evidence which found that grandparents were eating healthier and consuming more unprocessed, local foods compared to their adolescent grandchildren (41).

Parents were concerned about the increased preference for processed foods of their children, and as recent research from Kenya showed, they need to balance their parenting related to food between tradition and the modern realities of daily life (42).

With regard to socio-cultural dynamics, an important theme was overeating in a social context. A sign of a gracious host was to provide an abundance of meat and energy-dense foods with a high value placed on such foods. Meat and energy-dense foods were perceived as an indicator of high social status whereas vegetables were seen as an indication of poverty (22). Studies showed that consumers in high-income countries but in lower socioeconomic positions tended to eat more meat than consumers of higher socioeconomic backgrounds (43), which might be driven by the desire for a higher social status (44). In addition to meat consumption, our review found that large body size was indicative of higher social status. A review of African studies also described the association between larger body size and social standing as well as the importance of gaining weight after marriage symbolizing that the woman is well-cared for (45). Our review also found this with regard to the body size of the husband and children associated with how well a woman was perceived to take care of them (22, 30).

Gender roles were also influencing dietary behaviors, especially of women. In the included studies, men were encouraged to consume more food and were given priority of higher quality foods than women due to men historically being the breadwinner of the household, performing hard manual labor on the farm. Prioritizing men's food consumption affects women's dietary decision-making power and has also been shown to change women's dietary behaviors by adapting their diets to their husband's preferences (41). Having to fulfill their expected roles in the household and family while also working outside the house was shown to limit women's time to prepare food at home, making families more depended on processed food (22, 30, 37). However, no studies in this review focused on women's diets related to the workplace, which could have offered valuable insights into possible interventions for women given their increasing presence in the workforce. These types of time limitations have been associated with obesity in women (46).

With regard to the food environment, we found an increased reliance on processed foods, especially in urban settings where access to land is more limited reducing the accessibility of homegrown foods. However, studies in our review found that rural settings have also seen an increase in ultra-processed food consumption with improved infrastructure, and limited access to land with only 75% having access to food produced for home consumption (32). Fiji has seen a nutrition transition from traditional homegrown foods to modern highly processed imported foods with a 28% increase in processed foods sales between 2004 and 2018 (6). This nutrition transition in Fiji, and other low- and middle-income countries, has been driven by the movement of transnational food and beverage companies into emerging markets such as Fiji (1) and the globalization of food trade with several ultra-processed foods now playing a key role in Pacific diets. Studies on food imports found an increase in imports of healthy as well as unhealthy foods and beverages such as SSBs over 14 years in Fiji (6). Ravuvu et al. suggest that these changes in food imports are related to Fiji's membership to the World Trade Organization in 1995, which led to increases in tariffs on healthy foods from 2000 to 2010 and an increase in the import of highly processed and energy-dense foods between 2000 and 2010 (47). These food imports affect availability but also the affordability of food and are a phenomenon occurring across all the Pacific nations (6). In the Federal States of Micronesia for instance nutritious food items, such as tuna, are exported to high-income countries, while it has become unaffordable to the local population (48). The nutrition transition has led to unhealthier diets in the Pacific Islands (49), which in turn may have contributed to higher rates of overweight and other non-communicable diseases (50). In addition to the effects of the globalization of trade, the proliferation of the mass media has influenced the dietary intake of individuals in Fiji. Exposure to social mass media was reported to negatively impact eating behaviors in adolescent Fijians, leading to pathological eating behaviors associated with eating disorders (51).

Concerns about chemical or pesticide contamination of local food but also chemicals in imported food were reported in this review (24, 30, 31). These concerns related to the consumption of vegetables grown on land treated with pesticides may subsequently cause individuals to shift purchasing and consumption behaviors away from fruit and vegetables and toward ultra-processed foods due to their perceived hygienic packaging (52). Food safety issues place a large burden on the Pacific (53). More than 125 million people in the Pacific region fall ill from unsafe food, and more than 50,000 die (53). However, in a low- and middle-income country setting such as Fiji concerns regarding food safety may outweigh those of the nutritional properties of food, as was reported by a review on food safety concerns (52). These food safety concerns could overshadow or even distract from equally pressing issues related to the dietary transition such as increasing rates of overweight and obesity (54).

Environmental factors identified in this review were related to climate change and how it affects agricultural production. We found that Fijians were concerned about climate change, due to its impact on local food production and the interference with the food supply chain. Climate change is a pressing issue affecting the whole world but is particularly evident and immediate in Fiji with its low elevation and rising sea levels by 5.5 cm between 1992 and 2009 (55), increasing natural disasters such as cyclones, floodings, and droughts, and unpredictable seasons (56). The WHO cited malnutrition as a key climate-sensitive health risk alongside NCD-related illnesses, psychological impacts, and decreased access to health services (57). Studies included in this review reported that climate change has made it harder for Fijians to plant and grow crops which subsequently leads to a reduction in local food production. This increased reliance on imported, processed foods has a negative impact on individuals' dietary intake whilst simultaneously causing a greater economic burden, especially if any interruptions to the food chain occur due to climate change. Individual food choices can also influence climate change through changed demand for (un)sustainable foods, food waste, or social movements to mitigate climate change (58). However, our review could not find evidence of individuals' dietary choices and their influence on climate change, nor individuals adapting their diets due to concerns about climate change.

### 4.1. Research implications

This review identified only 22 studies assessing influencing factors of diets with only a few studies comprehensively examining multiple factors. Most of the studies assessed only dynamics at the individual or family level. There is a need for more studies assessing the underlying reasons for dietary behaviors in Fiji, especially related to the food environment. The few studies assessing how food supply or food environments influence diets relied on respondents' perceptions of food availability, promotion, and affordability. More research at the local level is needed using valid and reliable measures and study designs that allow for multi-level assessment of relationships between what kinds of foods are available, promoted, and affordable in people's food environments and their dietary behaviors. This could be achieved through quantitative and qualitative analysis of the personal and external food environment. The quantitative analysis could be achieved through mapping of vendors such as by using Geographical Information System mapping and qualitative analysis by interviewing caregivers to better understand people's lived experience in the food environment (59). Mixed-methods approaches are needed to link quantitative methods assessing actual food availability, prices, and advertising with the lived realities of residents in different neighborhoods (60).

### 4.2. Programme and policy implications

This review identified that Fijians largely understood what constituted a healthy diet and the importance of following a healthy diet to prevent non-communicable diseases such as obesity and diabetes. However, there was a disconnect between knowledge of what constituted a healthy diet and the consumption of healthy foods in practice, due to deeply ingrained cultural norms that promote the consumption of an unhealthy diet. Social and Behavior Change Communication strategies are needed to address these cultural and social norms related to the high status of energy-dense foods or meat, the pressure to overeat at social events as well as traditional gender roles, and intrahousehold food distribution.

Furthermore, policy actions should take climate-sensitive approaches, by revitalizing traditional farming practices, experimenting with salt and drought-tolerant crops or other innovative climate strategies, and building on the experience of small island states (56).

Policy actions should also ensure the availability of safe and affordable food by assessing trade policy commitments and strengthening risk-based food safety policies that contribute to the availability, nutritional quality, and safety of the food supply and help individuals make informed judgements about food safety hazards (47, 61).

### 4.3. Strength and limitations

Our review followed a rigorous review methodology for rapid reviews (12), searching three databases to identify relevant peer-reviewed quantitative, qualitative, and mixed methods studies. However, limiting the search to databases for published literature might have missed research from a Master's or PhD thesis that was not published. In addition, our study provides a good overview of the individual, social, and food environment dynamics influencing dietary behaviors in Fiji. However, as the study focused solely on Fiji, these results cannot be generalized to other Pacific Islands.

## 5. Conclusion

This rapid review identified factors at individual, social, and food environment levels influencing the dietary behaviors of Fijians, and also evidence gaps especially with regard to the food environment, calling for an integrated approach to research and programming to address these issues more systemically.

## Author contributions

SG, BB, and UT defined the scope of the review. UT conducted scoping searches to inform the search strategy. BB and UT conducted the search, screening, data extraction, and conducted the data synthesis. SG served as a reviewer for verifying data extraction. BB, UT, AN, and VL wrote the first draft of the article. All authors advised on the search, data extraction, analysis format, and read and approved the final article.
